# Where do our preferences come from? How hard decisions shape our preferences

**DOI:** 10.3389/fnbeh.2022.956307

**Published:** 2022-10-20

**Authors:** Katharina Voigt

**Affiliations:** ^1^Turner Institute for Brain and Mental Health, Monash University, Clayton, VIC, Australia; ^2^Monash Biomedical Imaging, Monash University, Clayton, VIC, Australia

**Keywords:** preference, decision-making, choice-induced preference change, preference formation, value-based decision model

## Introduction

Where do our preferences come from? Traditional neurocognitive models of value-based choice view decision-making as a serial process in which stable preferences are the basis of subsequent choices (Dolan and Dayan, 2013). An alteration of preferences is only expected if new (external) information about choice alternatives becomes available (e.g., through the consumption of a good). Accordingly, in a supermarket we assign values to items based on our stable preferences and choose the item we assigned the highest value to. After we tasted our selection, we can adjust our preferences for that item based on this recent experience. However, one highly debated question over the past decade has been whether preferences can change endogenously, that is, in the absence of any additional external information about the choice options, and merely as a function of our past choice history. Specifically, a growing body of studies found that when individuals must make binary choices between items they initially indicated to prefer equally well, their preferences for the chosen option increases and decreases for the rejected option. This empirical observation is now commonly referred to as the choice-induced preference change effect (reviewed by Izuma and Murayama, 2013; Enisman et al., 2021).

Prominent explanations of the choice-induced preference change effect are based on (Festinger, 1957) theory of cognitive dissonance, which proposes that discrepancies between actions and preferences cause psychological discomfort. Preferences are then adjusted *after* a hard decision has been made to reduce the dissonance between initial preference and the decision outcome (reviewed by Harmon-Jones et al., 2015). This explanation is in line with neuroimaging studies, which suggested that at the time of re-evaluation, after dissonance between preferences and choices is detected by the anterior cingulate cortex (ACC; van et al., 2009; Kitayama et al., 2013), the dorsolateral prefrontal cortex (dlPFC) triggers changes in the neural representation of value (Izuma et al., 2010, 2015; Mengarelli et al., 2015) in the ventromedial PFC (vmPFC) or ventral striatum (vStr; Izuma et al., 2010; Chammat et al., 2017). However, what happens in situations when we equally prefer two choice alternatives and therefore existing preferences are not sufficient to differentiate among them? In other words, how do we solve hard decisions?

An alternative possibility is that preferences are adjusted much earlier, that is, while a hard decision is being made, when the value differential of the options is not sufficient to choose among them. As such, preference adjustments might constitute a necessary adaptive (online) mechanism to deal with hard choices, as opposed to a post-decisional process for eliminating cognitive dissonance (Izuma et al., 2010, 2015). This new hypothesis, however, remains largely untested as existing functional neuroimaging studies focused entirely on the neural mechanisms of preference change during re-evaluation (Izuma et al., 2010; Chammat et al., 2017). Studying decisions among equally preferred items, however, holds key in understanding how our preferences are dynamically constructed based on the choice context.

Our recent neuroimaging study is the first to study preferences changes during hard decisions and provides evidence for this alternative theory of choice-induced preference changes (Voigt et al., 2019). Preference changes were predicted from activity in left dorsolateral prefrontal cortex and precuneus while making hard decisions. Fixation durations during this phase predicted both choice outcomes and subsequent preference changes. These preference adjustments became behaviorally relevant only for choices that were remembered and were in turn associated with hippocampus activity. These findings suggest that preferences evolve dynamically as decisions arise, potentially as a mechanism to prevent stalemate situations in underdetermined decision scenarios. Based on this recent evidence from neuroimaging I propose a novel neural framework of choice-induced preference changes, which is described in the following.

## An integrative neural model underlying endogenous preference formation during hard decisions

**Figure 1:**
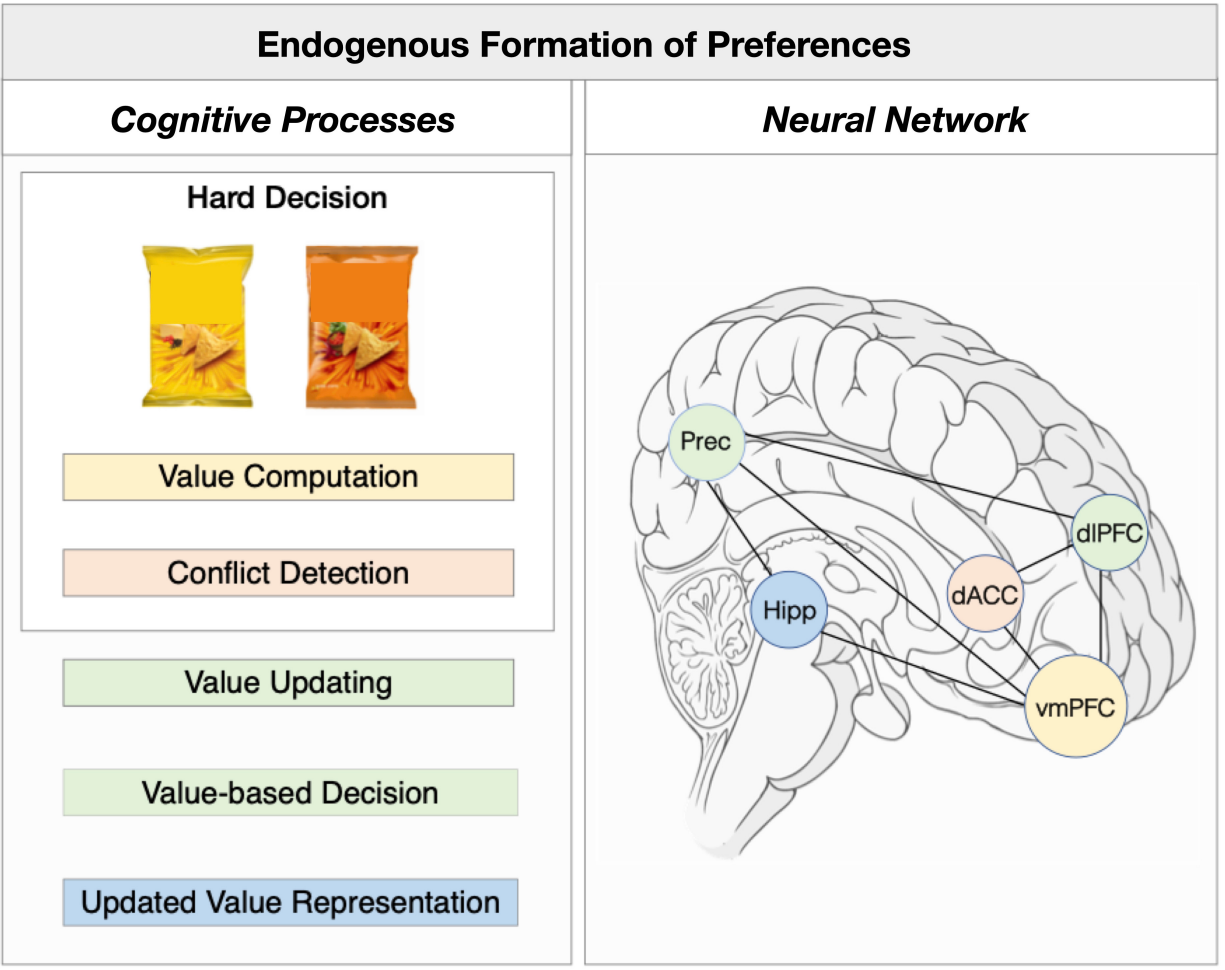
Integrative neural model underlying endogenous preference formation. Left Panel: The underlying cognitive mechanisms of choice-induced preference change effects can be described into four main processing steps: (1) value computation (orange) (2) conflict detection (red), (2) value updating and (3) value-based decision (green), (4) updated value representation (blue). Right Panel: Neural regions and networks associated with the cognitive processing steps of choice-induced preference change effects. Colors of cognitive processes match the neural correlates as depicted in the neural processes. Hipp, Hippocampus; Prec, Precuneus; dACC, dorsal anterior cingulate cortex; vmPFC, ventromedial prefrontal cortex; dlPFC, dorsolateral prefrontal cortex.

In the next section i will integrate recent empirical evidence that supports an alternative model of choice-induced preference effects. This tentative, integrative neural process model describes endogenous preference formation during hard decisions in four processing steps: (1) value computation (2) conflict detection, (2) value updating, (3) value-based decision, (4) updated value representation (Figure 1).

**(1) Value computation:** The first process involves the computation of value for both choice alternatives. This computation facilitates a comparative process, allowing the decision maker to identify and pursue the option with greatest expected value (Samuelson, xbib1938). Value computation is further essential in triggering the preparation of upcoming motor responses: to make appropriate decisions, these values must be reliable predictors of the benefits that are likely to result from each action. There is converging evidence that value computation is predominantly associated with prefrontal areas, such as the vmPFC (Kable and Glimcher, 2007; Chib et al., 2009; Bartra et al., 2013), dlPFC (Hare et al., 2009; Sokol-Hessner et al., 2013), but also the vStr (Bartra et al., 2013). The vmPFC in particular has been shown to compute value as a ‘common currency’ (Chib et al., 2009) and its activity is related to upcoming computations of motor choice responses (Rudorf and Hare, 2014).

**(2) Conflict detection:** If the result of value computation is that the value differential between both items is not sufficient to distinguish between the choice items, and therefore, no motor preparations can be triggered, a moment of indecision or decision conflict occurs. If no decision conflict is detected, that is, the value differential is sufficient to discriminate among the options (e.g., during easy decisions), then the system can compute a decision value and continues with the value-based choice (as described in the fourth step of this model). Behavioral evidence suggests that preference changes do not occur for easy choices, but only for hard decisions and individuals take significantly longer to solve hard over easy decisions (Voigt et al., 2019). Neuroimaging studies revealed that hard decisions, compared to easy decisions, were associated with dACC activity (Kitayama et al., 2013; Voigt et al., 2019). Previous studies linked activity in the dACC to decision conflicts (Kitayama et al., 2013; Shenhav et al., 2013; Shenhav and Buckner, 2014).

**3) Value updating:** Fixation duration plays a causal role in value-guided choice (e.g., Shimojo et al., 2003; Glaholt et al., 2009). Krajbich et al. (2010) showed that the fixation duration for an item mirrors the evidence accumulation process for an decision outcome. Eye-tracking data during the process of making hard decisions revealed that the total and first fixation duration for of an item was predictive of its choice and, importantly, its subsequent change in value (when controlling for choice) (Voigt et al., 2019). The latter finding gives reason to assume that fixations contribute to the construction process of new preference values prior to the choice. In the light of these and our findings, the proposed preference formation model implies that in underdetermined decision scenarios, the decision system extracts new information in the moment of choice via fixation on a particular item in order to construct new preferences guiding upcoming choices. This in turn might reflect an adaptive mechanism to solve hard decisions.

Activity in the dlPFC and precuneus was linked with online, trial-by-trial updates of preferences during hard choices (Voigt et al., 2019). The left dlPFC was previously shown to be involved in the implementation of preference change *after* the difficult choice was made (Izuma et al., 2010; Harmon-Jones et al., 2015; Mengarelli et al., 2015). Both the dlPFC and precuneus have been previously associated with dissociable roles in working memory (Brodt et al., 2016), shifts of spatial attention (Yan et al., 2016) and value reconstruction (Harris et al., 2011). Specifically, the precuneus was shown to be involved in the early bottom-up selection of spatial attention, whereby the dlPFC was associated with later top-down selection of spatial attention. As fixations were shown to play a role in preference reconstruction it is reasonable to assume that initially the precuneus, which has rich connections to the superior colliculus administering eye-movements (Yeterian and Pandya, 1998), allocates spatial attention to the salient stimulus (i.e., bottom up) and forwards this information to the dlPFC. Previous studies showed that the dlPFC ‘holds’ choice-relevant information in working memory (Brodt et al., 2016) in order to guide performances toward targets. This temporal dynamic value representation evolving from posterior to prefrontal regions was demonstrated in other studies (Harris et al., 2011). These findings suggest that initial bottom-up shifts in spatial attention are explained by in-decisional precuneus activity controlling the reconstruction of new value information, which is forwarded to the dlPFC. This representation is then stored into working memory assisting the individual to maximally discriminate between the choice options and implementing the choice.

**(4) Value-based decision:** Based on the rapid fixation-guided computation of a new value signal, which might be subserved by a network subtending the precuneus and dlPFC, the decision system is now able to compute a decision variable as the value differential among the alternatives is now sufficient to distinguish between them. This means, the decision scenario now has transferred from a hard to a (relatively) easy one. The computation of decision value has been associated with dlPFC activity, which is connected to premotor areas conducting the actual motor choice (Miller and Cohen, 2001). Further, transient disruption of human dlPFC induced by theta-burst transcranial magnetic stimulation has been shown to interfere with forward planning and flexible, outcome-specific decision behavior (Smittenaar et al., 2013). This functionality of dlPFC activity might also explain why previous research investigating choice-induced preference change effects found the involvement of the dlPFC at the stage of re-evaluation (e.g., Izuma et al., 2010, 2015). In the model, this might simply reflect the computation of an upcoming value-based choice, but not necessarily the update of value itself as it was previously proposed.

**(5) Updated value representation:** Although preference changes were updated during hard choices, behavioral evidence indicates that only choice outcomes that are explicitly remembered were encoded as preference changes long-term (i.e., at re-evaluation); choice outcomes that were forgotten or guessed did not trigger long-lasting preference changes (Salti et al., 2014; Chammat et al., 2017; Voigt et al., 2019). Neuroimaging studies showed that this memory-dependent choice-induced preference change effect is associated with left hippocampus activity (Chammat et al., 2017; Voigt et al., 2019). The hippocampus plays a key role in long-term and it has strong reciprocal anatomical connections with the vmPFC (Weilbächer and Gluth, 2017), supporting its role in long-term representations of endogenous preference changes.

## Conclusion

This opinion paper presented empirical findings that support the notion that our preferences evolve endogenously during the process of making decisions between equally preferred items. In those situations of indecision, the information gathered via fixation toward an item seems critical to reconstruct upcoming value-based decisions. This online mechanism of fixation-driven preference formation might be depended on the idiosyncrasy of an underlying prefrontal-parietal brain network. Such rapid changes in preferences prior to the initial undetermined decision, could constitute an adaptive mechanism enabling the individual to act. These in-decisional changes in preferences become behaviorally manifested when choice outcomes were explicitly remembered and encoded by episodic memory regions. Overall, these findings suggest a potential rethinking of the very notion of preferences and value-based choice. Further, they suggest a shift away from previous explanations of endogenous preference formation. Rather these findings suggest that seemingly self-determined, subjective cognitive concepts, such as our preferences, might be emergent consequences from the particulars of the decision scenario itself and brain networks underlying value-based decisions.

## Author contributions

The author confirms being the sole contributor of this work and has approved it for publication.

## Funding

This paper was funded by Dr. Rüdiger Seitz, via the Volkswagen Foundation, Siemens Healthineers, and the Betz Foundation.

## Conflict of interest

The author declares that the research was conducted in the absence of any commercial or financial relationships that could be construed as a potential conflict of interest.

## Publisher's note

All claims expressed in this article are solely those of the authors and do not necessarily represent those of their affiliated organizations, or those of the publisher, the editors and the reviewers. Any product that may be evaluated in this article, or claim that may be made by its manufacturer, is not guaranteed or endorsed by the publisher.
